# Supplementary material for: Non-Additive Effects of Environmental Factors on Growth and Physiology of Invasive Solidago canadensis and a Co-Occurring Native Species (Artemisia argyi)
Source: Plants (Basel). 2022 Dec 27;12(1):128. doi: 10.3390/plants12010128 (PMC9823473; doi:10.3390/plants12010128)

Supplementary Material

Non-additive effects of environmental factors on growth and physiology of invasive *Solidago canadensis* and a co-occurring native species (*Artemisia argyi*)

Bin Yang, Miaomiao Cui, Zhicong Dai, Jian Li, Haochen Yu, Xue Fan, Susan Rutherford*, Daolin Du*

Institute of Environment and Ecology, Academy of Environmental Health and Ecological Security, School of the Environment and Safety Engineering, Jiangsu University, Zhenjiang, 212013, China

Table S1 Comparison of plant traits of invasive *Solidago canadensis* and its co-occurring native species *Artemisia argyi*

	Solidago canadensis L.	*Artemisia argyi* H.Lév. & Vaniot
Family [1]	Asteraceae (Compositae)	Asteraceae (Compositae)
Genus [1]	*Solidago*	*Artemisia*
Life Form [1]	Herbaceous	Herbaceous or Subshrub
Growth Form [1]	Perennial	Perennial
Growth Community [2]	Temperate biome	Temperate biome
Native Distribution [2]	North America	East Asia
Introduced Distribution [2]	Asia, Europe, Oceania	North Asia, Central Asia, Middle East, Eastern Europe
Co-occurring Habitats in China [3]	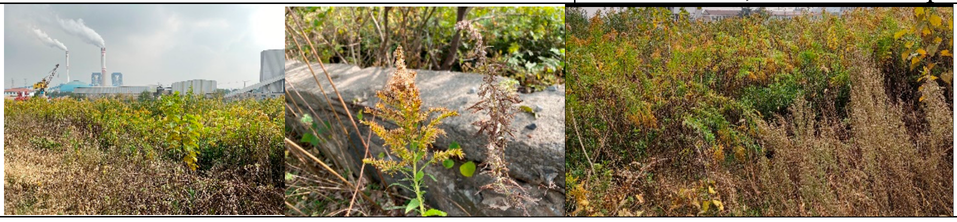	

1 FOC, Flora of China Available online: http://www.iplant.cn/foc (accessed on 30 November 2022).

2 Plants of the World Online | Kew Science Available online: https://powo.science.kew.org/ (accessed on 30 November 2022).

3 The photos were taken in the suburb of Zhenjiang City, Jiangsu Province, China (119.57548 E, 32.18547 N) in November 2021.

Table S2 Temperature differences between the treatments during the experiment showing paired t-test and correlation analysis

Temperature Data (mean ± SE, C°）			Paired t-test			Correlation Analysis		
Ambient Group	Warming Group	Warming Amplitude	*t*	*df*	*p-value*	*df*	R^2	*p-value*
29.13±0.25	31.36±0.25	+2.23±0.08	27.633	76	<0.001	77	0.948	<0.001

Figure S1 Relative interaction effect (RIE) of temperature and UV on plant performance in invasive *Solidago canadensis* and a co-occurring native species, *Artemisia argyi,* across plant communities (i.e., invasive species only, native species only and mixed community). RIE was calculated using the formula in Crain et al. **[25]**: $\boldsymbol{RIE} = \frac{(\boldsymbol{U}\times\boldsymbol{T}-\boldsymbol{CK})-((\boldsymbol{T}-\boldsymbol{CK})+(\boldsymbol{U}-\boldsymbol{CK}))}{|\boldsymbol{CK}|}$, where *CK* represents the ambient UV and ambient temperature treatment, *T* represents the ambient UV and warming treatment, *U* is the high UV and ambient temperature treatment, and *U* × *T* is the high UV and warming treatment. Values are the mean ± standard error (SE). 'AD' above the dots indicates that the interaction between temperature and UV is not significant (i.e., SE = 0, and is therefore an 'additive' effect), '+' indicates a synergistic effect (where SE is greater than 0), and '-' denotes an antagonistic (-) effect (where SE is less than 0). The charts on the right, show the proportion of traits in each species affected by additive, synergistic or antagonistic interactions between temperature and UV when grown in mono- and mixed culture.

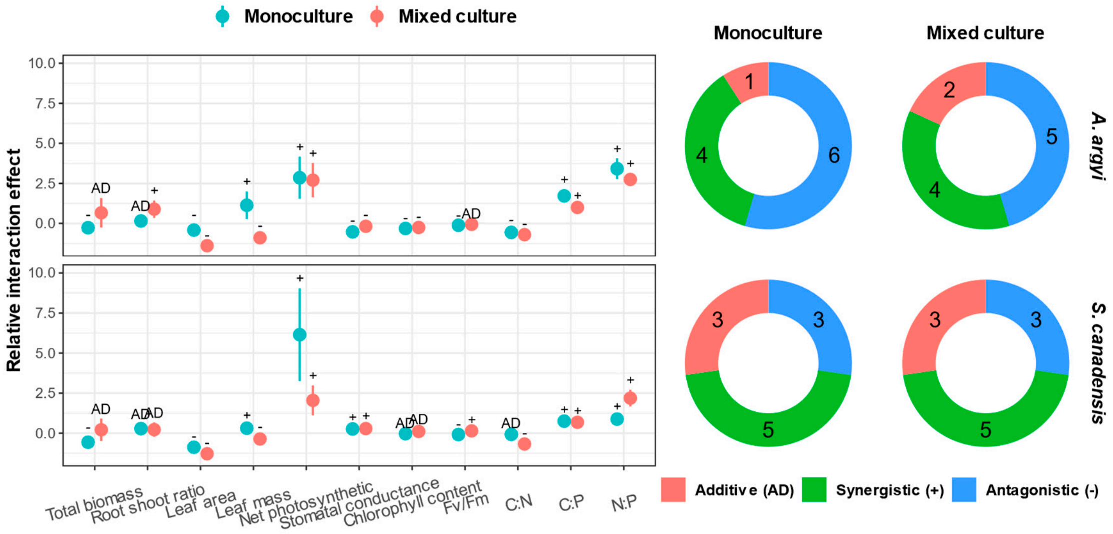

Supplement: Supplementary file 1 [file plants-12-00128-s001.zip › plants-2073555-supplementary.pdf]
